# Reversible Data Hiding in FTIR Microspectroscopy Images with Tamper Indication and Payload Error Correction

**DOI:** 10.1155/2017/7584852

**Published:** 2017-11-13

**Authors:** Angelos Fylakis, Anja Keskinarkaus, Juha Partala, Simo Saarakkala, Tapio Seppänen

**Affiliations:** ^1^Center for Machine Vision and Signal Analysis, University of Oulu, 90014 Oulu, Finland; ^2^MRC Oulu, University of Oulu, 90014 Oulu, Finland; ^3^Research Unit of Medical Imaging, Physics and Technology, University of Oulu, 90014 Oulu, Finland; ^4^Department of Diagnostic Radiology, Oulu University Hospital, 90029 Oulu, Finland

## Abstract

Fourier transform infrared (FTIR) microspectroscopy images contain information from the whole infrared spectrum used for microspectroscopic analyses. In combination with the FTIR image, visible light images are used to depict the area from which the FTIR spectral image was sampled. These two images are traditionally acquired as separate files. This paper proposes a histogram shifting-based data hiding technique to embed visible light images in FTIR spectral images producing single entities. The primary objective is to improve data management efficiency. Secondary objectives are confidentiality, availability, and reliability. Since the integrity of biomedical data is vital, the proposed method applies reversible data hiding. After extraction of the embedded data, the FTIR image is reversed to its original state. Furthermore, the proposed method applies authentication tags generated with keyed Hash-Based Message Authentication Codes (HMAC) to detect tampered or corrupted areas of FTIR images. The experimental results show that the FTIR spectral images carrying the payload maintain good perceptual fidelity and the payload can be reliably recovered even after bit flipping or cropping attacks. It has been also shown that extraction successfully removes all modifications caused by the payload. Finally, authentication tags successfully indicated tampered FTIR image areas.

## 1. Introduction

Capturing image information from the whole infrared spectrum limited only by the near diffraction limit of light has now allowed scientists to further exploit the possibilities of microspectroscopic analysis. To give an example, analyzing the infrared spectrum enables the acquisition of biochemical information out of a tissue for the diagnosis and the assessment of cell functionality [1, 2]. Specifically, Fourier transform infrared (FTIR) microspectroscopy is a widely used technology in life science applications. In FTIR microspectroscopy, a FTIR spectrometer is connected to an optical microscope and the transmission of infrared light through the sample (or the reflection from the sample surface) is measured as a function of wavelength. Standard FTIR microscopes can collect chemical microscopic images with a spatial resolution of approximately 5–10 *μ*m [3]. FTIR microspectroscopy helps scientists to study and analyze the heterogeneity of samples, their biochemical characterization, and, for example, the distribution of drugs in tissues. Nowadays, storage and circulation of FTIR image files become more and more common and thus new requirements come to place. Those requirements have to do primarily with the efficient management of this data and secondly with security issues. In this paper, we will specifically deal with those requirements using FTIR spectral images and their corresponding visible light images sampled from the human articular cartilage. Currently, those two images are transferred and stored in databases as separate files.

Data management efficiency refers to storing and transferring multiple data entities as efficiently as possible. In this case, by multiple entities, we refer to the FTIR images and their corresponding visible light images, as well as other possible Electronic Patient Records (EPR). Because of regular transfer of biomedical material, there is a risk of data loss. Constant transfer and storage of biomedical data raises other security issues. Some among the most important are data confidentiality, availability, and reliability [4]. Concerning confidentiality, biomedical data can come with private patient information and thus direct access to all data by nonauthorized users would violate the privacy. Availability refers to the ability to access all the different entities or files at any moment. This is not necessarily guaranteed when data is stored and circulated in separate files. Last, reliability is related to the problems of verifying integrity of data, as well as tracing and validating authentic data. Tampered data can mislead and cause errors in the diagnosis and similar problems can occur when nonauthentic material is used. Since biomedical data is sensitive and sometimes used even for diagnoses, mechanisms and techniques for tracing and validating authentic content are of high importance. A data hiding method is a complementary solution to the management and security issues by enabling the combination of different data entities in one, as well as the recoverability of the payload and the tamper proofing of the host data. Since in this case we use biomedical data, a reversible data hiding method is preferred. Reversible data hiding is described as the process of embedding a set of data *w* in a host digital object *I*. This process produces a new object *I*_*w*_ from which *w* can be reliably located and extracted [5]. Parallel to extraction, *I*_*w*_ is reversed to its original state *I* by removing all modifications.

This paper proposes a data hiding method for embedding the visible light image in the FTIR spectral image, including authentication tags of the original FTIR image generated using Hash-Based Message Authentication Codes (HMAC) for authentication and tamper proofing purposes. Moreover, all necessary side information is also included in the payload. Prior to embedding, all payload data is encoded using error correcting codes. Because of the sensitivity of the host, after the extraction of the payload from the FTIR image, all modifications are removed and therefore its original state is recovered. The error correcting codes used in this method were designed to recover data from two attack scenarios. The first one is least significant bit flipping due to noise and the other one is removal attacks. Both attacks might take place in one, more, or all spectral components of FTIR pixels. All experiments in this paper used research data collected from Oulu university hospital taken from human articular cartilage samples, that is, the connective tissue that provides resistance to compressive forces during joint movements [2].

In this method, by combining all the entities associated with the FTIR microspectroscopy in a single package, data management efficiency is increased since the access to the different entities is done through a single FTIR image. Furthermore, storing all components in the same structure reserves more space and the linkage between files is not necessary any more as everything is included in this same structure. Access to embedded data is only given to entitled users with access to the extracting code. Furthermore, all information such as patient details or EPR can be encrypted before it is hidden and therefore confidentiality is maintained. Availability is guaranteed since all the data is combined in the same entity and thus access to the visible light image for a given FTIR segment is always available. Last, reliability is assured since tampered areas can be revealed using authentication tags. To sum everything up, for data management efficiency and security purposes, this paper proposes a method of embedding related data in FTIR spectral images, including authentication tags. This enables constant availability of data and tamper detection capabilities.

The paper is organized as follows.  Section 2 includes the state of the art in hyperspectral image watermarking. Past literature is primarily dealing with hyperspectral satellite images. Further to that, some biomedical applications based on the comparable data are also presented.  Section 3 features the key technological components used in this method.  Section 4 includes the main part of this paper, that is, the description of the data hiding methods.  Section 5 explains the data formats concerning the FTIR spectral image and the visible light image. Secondly, it presents the experimental results and the discussion. Last, Section 6 is the conclusion part of this paper, including suggestions for improvements on future work.

## 2. State of the Art

Before the reference to biomedical applications, this section begins with the background of hyperspectral image data hiding and watermarking. Hyperspectral imaging concerns the collection and process of information originating from the whole electromagnetic spectrum. The purpose of hyperspectral imaging is to obtain the spectrum for each pixel in the image of a scene in order to find objects, identify materials or tissues, and detect processes [6, 7]. Past data hiding literature focuses on remote sensing satellite images. Remote sensing is a subfield of geography. In modern usage, the term generally refers to the use of aerial sensor technologies to detect and classify objects on Earth. The data is sampled from multiple parts of the electromagnetic spectrum and combined with larger scale aerial or ground-based sensing. Consequently, it provides researchers with enough information to monitor trends, including both long and short term phenomena. Data hiding in 3D hyperspectral structures can be done with two different approaches. Firstly, it is done by processing each pixel independently. In this case, though, the size of the vectors might be too small; thus the redundancies are not enough to give enough capacity to store the watermark. Secondly, it is possible to process each image row as a 2D image structure, the second dimension given by the wavelength. This corresponds to the most usual capture process.

In 2003, Tamhankar et al. [8] proposed a watermarking scheme based on the Redundant Discrete Wavelet Transform (RDWT) and applied to hyperspectral signatures to protect ownership rights and deter any illegal use. It is designed for the data collected by handheld devices that measure reflectance of electromagnetic waves in the wavelength range of 350 nm to 2500 nm. For embedding, the watermark the RDWT was chosen as the transform domain. Specifically, the watermarks were hosted in the RDWT coefficients from the second level of two-level decomposition.

In 2003, Kaarna and Toivanen [9] developed a hyperspectral image digital watermarking method where the payload is a grayscale image. The method is based on the use of Principal Component Analysis (PCA) transformation getting the eigenimages and eigenvectors and in a second stage the wavelet transform is applied to each eigenimage. Each pixel of the wavelet transform of the watermark image is embedded in the most appropriate eigenhost image transform. In that way, the watermark is embedded throughout the spectrum. It is a well considerable approach when robustness against noise or lossy compression is required. In 2004, Kaarna and Parkkinen [10] proposed an improved version where the multiwavelet transform is used. Specifically, the method uses the Chui-Lian multiwavelets [11] to create the 3D wavelet transform of the host hyperspectral satellite image. As for the payload, it is again a grayscale image. For the watermarking process, first, a single level scalar wavelet transform is produced from the watermark image. Then, the 3D multiwavelet transform is generated from the hyperspectral host image and the watermark is added. For each watermark pixel, the most appropriate band of the transform block is selected. In 2004, Qin et al. [12] presented a semifragile watermarking scheme based on wavelet transforms. The edge and texture of the remote sensing image are extracted and the watermark is embedded only in the edge character. In that paper, only one band is marked. One of the purposes of the watermarking technique is to also provide information for tampered areas.

In 2006, Kbaier and Belhadj [13] proposed a Discrete Wavelet Transform- (DWT-) based watermarking scheme for remote sensed multispectral images. For reduced Mean Square Error (MSE) figures, they calculated the embedding strength separately for each spectral band. Serra-Ruiz and Megías in 2010 [14] proposed a method that uses the whole signature to embed a watermark for tamper detection using vector quantization. In the same year, Serra-Ruiz and Megías [15] proposed a semifragile watermarking scheme for remote sensing images. They exploit DWT watermarking and the low frequency (LL) subband in order to offer tamper proofing capabilities. The watermarks' fragility is exploited to indicate tampered image areas. The method can be tuned to embed the mark according to band relevance, depending on the content and the signatures to be protected. The technique is robust on JPEG compression and fidelity was high for every band, that is, PSNR over 60 dB.

The literature review did not reveal any hyperspectral watermarking techniques proposed for biomedical data. Nevertheless, reversible data hiding for data tampering and recoverability is considered by Liew and Zain [16]. The paper presents a method to protect 8-bit grayscale ultrasound images from unauthorized modification or destruction. The method is based on LSB substitution and on the Region of Interest-Region of No Interest (ROI-RONI) concept. Authentication and recovery data replace LSBs of the ROI, and, to enable reversibility, bits that have been replaced are embedded in RONI area that does not require reversion.

Other techniques more similar to FTIR spectral image data hiding technique are biomedical video watermarking methods. That is because the FTIR structure is similar to the video, but instead of the time dimension of the video, the third dimension is the one going over spectral components. Two examples are the data hiding method proposed by Dey et al. in 2012 [17] and by Achrajee et al. in 2014 [18]. The papers present data hiding methods designed to embed EPR or other data in intravascular ultrasound video. Both methods rely on redundancy in the motion vector where the watermark bits are embedded.

## 3. Key Technological Components

The proposed data hiding method makes use of a few key technological components. Some of those are established methods like those used for error correction and others were developed specifically for use on the currently proposed data hiding method. This section gives the necessary information about them to clarify the step by step description of the data hiding method in the next section.

### 3.1. Hash-Based Message Authentication Codes (HMAC)

The data hiding method includes the payload authentication tags created using HMAC. Those are generated from the original FTIR before the data is inserted. After extraction and reversion, authentication tags are generated again from the FTIR and compared with the ones that have just been acquired from the extracted payload. This comparison indicates whether some locations or spectral components of the FTIR have been possibly tampered. If there are no such indications, it confirms that reversion runs successfully and that the modifications of hidden data have been removed. Furthermore, they can indicate tampering that was caused because of noise or other forms of attack in the host FTIR spectral image.

In cryptography, a keyed Hash Message Authentication Code or, for short, HMAC, is a specific type of message authentication code (MAC) involving a cryptographic hash function (hence, the “H”) in combination with a secret cryptographic key. They are used for data integrity and authentication of messages.

Cryptographic hash functions are typically used to ensure the integrity of data. A cryptographic hash function can be applied on a message of arbitrary length to generate a short fixed sized message digest that can be subsequently used for integrity testing. MACs are keyed cryptographic hash functions that additionally guarantee authenticity of the data. For a MAC, a secret key is needed in order to generate a valid digest. The same key is also needed to check the validity of the digest.

In our case, both the data and the message digest are embedded, which means that a hash function is not sufficient (an attacker could easily modify both the data and its digest). We apply a keyed HMAC using the SHA256 hash function to ensure the authenticity of the data. HMAC [19] is a well-studied and used method of turning cryptographic hash functions into MACs with a proof of security [20]. SHA256 is a member of the SHA-2 family of cryptographic hash functions [21] with a 256-bit digest length. It is one of the most widely used cryptographic hash functions and widely considered to be secure.

HMAC-SHA256 is, in this case, used to create unique identifiers, that is, authentication tags for bundles of spectral components, as well as groups of pixels to locate spatial and spectral location of tampered data.

### 3.2. Reed-Solomon Codes

This paper is going to analyze two basic threat scenarios. The first one included attacks that caused bit flips and the second one cropping and removal attacks causing missing bits. For the first scenario, Reed-Solomon codes are the most appropriate solution.

Reed-Solomon codes are a group of error correcting codes introduced by Reed and Solomon in 1960 [22]. It is a widely used method for error correction in data disks. Reed-Solomon codes are specifically designed to correct multiple symbol errors. It is a linear block code of length *n* with dimension *k* and minimum Hamming distance *n* − *k* + 1. It will be represented with* ReedSolomon*(*n*, *k*) and it can correct up to (*n* − *k*)/2 symbols. For instance, a Reed-Solomon code operating on 8-bit symbols has *n* = 2^8^ − 1 = 255 symbols per block.

### 3.3. Deletion Channel Correction

The second threat scenario that will be analyzed in this paper is the one with cropping or removal attacks. In this case, since we are using FTIR spectral images, one, more, or all spectral components are removed out of certain pixels. Due to this process, it is most likely that bits will be missing out of the extracted bit stream. The end result is a deletion channel.

For the deletion channel correction, the proposed method uses the implementation by Duda using correction trees. The correction trees are big trees that contain all the possible solutions. The encoding method that was used of this implementation is based on encoding used in binary symmetric channel in the past [23].

This scenario may have exceptions, including bit flips instead of missing bits. Those errors can be accepted in most of the payload data but not in the side information. A single error in the extracted side information necessary in each iteration of the extracting procedure can cause it to halt and therefore a solution had to be designed. This solution was based on a time-consuming trial and error approach and thus this is why it was only applied for side information and not for the whole payload. Given a chunk of data, what the method does is detecting the existence of bit errors that the deletion channel correcting code is unable to repair. Then, it attempts to proceed by removing parts of this chunk until the problematic bits are removed and replaced by the original ones. This relies on the fact that the problem has been converted to a deletion channel.

### 3.4. Histogram Shifting

**Figure 1:**
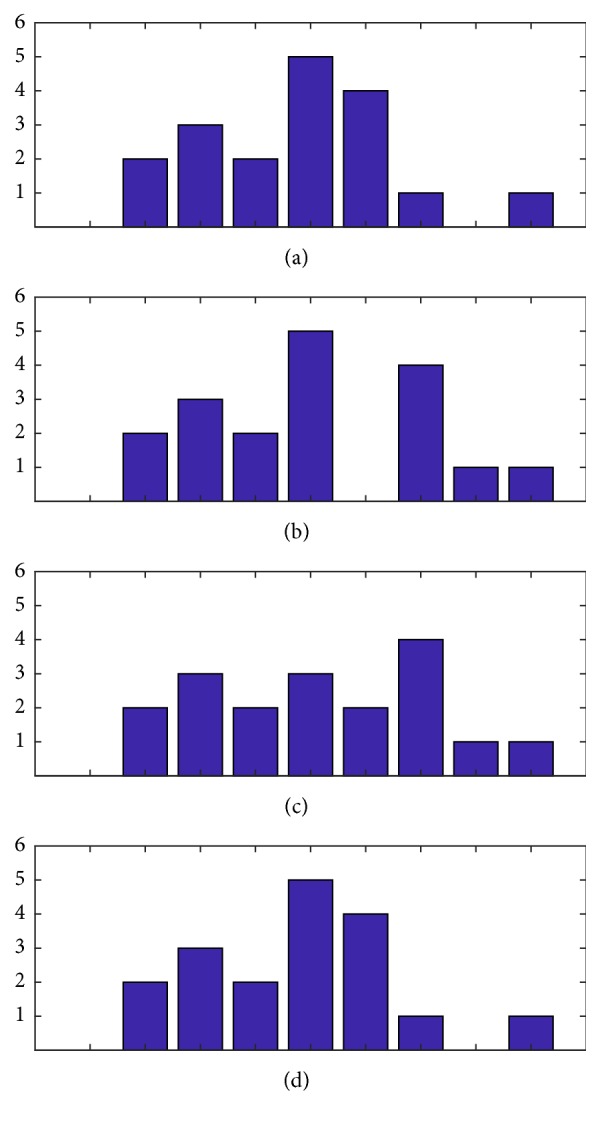
(a) The original histogram. (b) The histogram as a result of shifting to the right the segment for each index *i*, peak < *i* < zero, where peak is the index of the peak histogram point and zero is the index of the zero point. (c) The modified histogram. (d) The reversed histogram.

Reversibility and high capacity were the most important requirements in his method. Therefore, a histogram shifting-based approach was preferred. This concept was introduced by Ni et al. [24] followed by improvements by Tsai et al. [25] and Fallahpour et al. [26]. The idea behind this technique is that a zero and a peak point are located in the image histogram and then by modifying corresponding pixel intensities all values between the peak and zero point are shifted towards the zero point creating one empty bin next to the peak. Then, once again, the image is scanned pixel by pixel and pixels with intensity value corresponding to the peak point are lowered or increased to match the value of the emptied bin. Modified values represent 1 s and unmodified values represent 0 s. After extraction, the histogram is shifted back to its original state reversing also the image to its exact initial state. The process is illustrated in Figure 1.

Tsai et al. [25] implemented a data hiding method designed for static biomedical images. In the proposed scheme, histogram shifting data hiding was applied after employing a linear prediction technique on the host image to expand linear prediction errors instead of pixel intensities. First, the image is divided into *n* × *n* blocks; then in each block a basic pixel is selected. The difference between the basic and the other pixels is calculated and replaced with the original values calculating the residual image. Last, histogram shifting is applied with one or more peak and zero pairs. Similarly, Zeng et al. [27] implemented a reversible data hiding method in uncompressed videos exploiting their interframe prediction. This method performs double histogram shifting (two peak and two zero points) in the histogram produced from the estimation error for each video frame as follows:

(1) Eim,n=Fim,n−Fi′m,n,∀m,n∈N,  m∈1,M,  n∈1,N,

where *F* represents the current frame, *F*′ the predicted frame, and *M* × *N* their dimensions. The method starts embedding from the last frame to the first and, in each iteration, the detected peak and zero points are embedded fused with the payload in the next pair of frames to be used as side information in the extracting procedure. When the last frame is reached, an approach without the need for a reference frame can be used to store the last bits of the peak and zero points. In the extracting procedure, the loop is performed the other way around so that the side information for the next frame would always be available. The basic idea was reworked for FTIR spectral images. The inspections showed suitability of using the difference matrix between two consecutive spectral components as follows:

(2) Esm,n=Ism,n−Is−1m,n,∀m,n∈N,  m∈1,M,  n∈1,N,

where *I*_*s*_ and *I*_*s*−1_ are the two spectral components and *M* × *N* the dimensions of every component.

### 3.5. Data Quantization

In digital images, in order to form digital function, the gray-level values have to be converted into discrete quantities. This process of assigning the intensity levels to discrete values is called quantization [28]. The quantization process can also refer to downsampling discrete values. The current implementation falls in the second case as initially the quantization step was low due to the large bit depth and accordingly the generated histogram did not provide enough capacity.

The tested FTIR image samples were in 24-bit floating-point format, which for the difference matrix of two spectral components means 2 × 2^24^ − 1 histogram bins, while the original histogram shifting method was optimized for 8-bit images and thus for 2 × 2^8^ − 1 bins. For this reason, similar to our previous research paper [29], the solution was to downsample the FTIR spectral component difference matrix histogram from 2 × 2^24^ − 1 to 2 × 2^8^ − 1 bins. This downsampling process should not downgrade the actual quality; otherwise, reversion is not possible. For this reason, what is applied is not actual downsampling but a process better described as “grouping.” The difference matrix is not modified but instead a histogram is generated with histogram bins corresponding to a broader range of values. The quantization step is calculated as follows:

(3) $$\begin{array}{c} Q=\frac{2\left(\underset{}{\max}v-\underset{}{\min}v\right)+1}{bns}, \end{array}$$

where $\underset{}{\max}v$ is the maximum intensity FTIR value, $\underset{}{\min}v$ the minimum intensity FTIR value, and bns the target number of histogram bins. In our case, data quantization enables the creation of discrete histograms ranging in [−255, 255] as they would have done in regular 8-bit images. In this way, the capacity is also highly increased being equal to the sum of the two highest peaks in the histogram. Furthermore, robustness is also considerably increased with the proposed method.

## 4. Proposed Method

This section describes the proposed reversible data hiding method step by step. The proposed method includes the two basic data hiding procedures, that is, the embedding procedure, where the payload is hidden in the FTIR spectral image, and the extracting/reversing procedure, where the payload is extracted and the FTIR image reversed.

### 4.1. Overall Description

**Figure 2:**
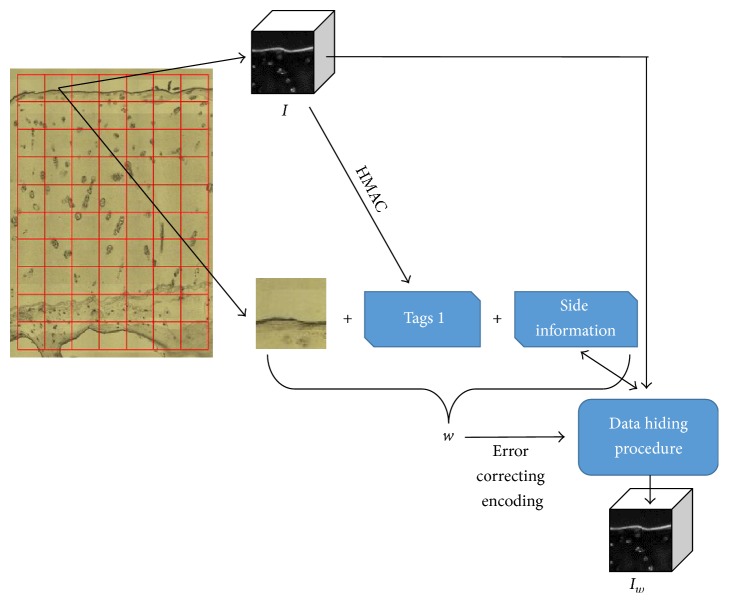
Embedding the payload *w* in host FTIR image *I*.

The payload *w* is embedded in the FTIR spectral image *I* with imperceptible modifications producing *I*_*w*_. This payload *w* includes the following four components: (1) the necessary side information required by the histogram shifting technique that includes the two peak and zero locations at the histograms; (2) the equivalent visible light image, a regular RGB image depicting the area from which the FTIR image was sampled; (3) the authentication tags produced from the original FTIR image *I* useful for showing possible tampering at the FTIR image; and (4) other optional possibly private data or patient information as, for instance, the Electronic Patient Records (EPR). Before the payload *w* is embedded in the host, it is encoded using error correction codes to make it recoverable even in the case of attacks that might later occur in the host. The embedding procedure for a block chosen out of the grid on the left is demonstrated in the block diagram in Figure 2.

**Figure 3:**
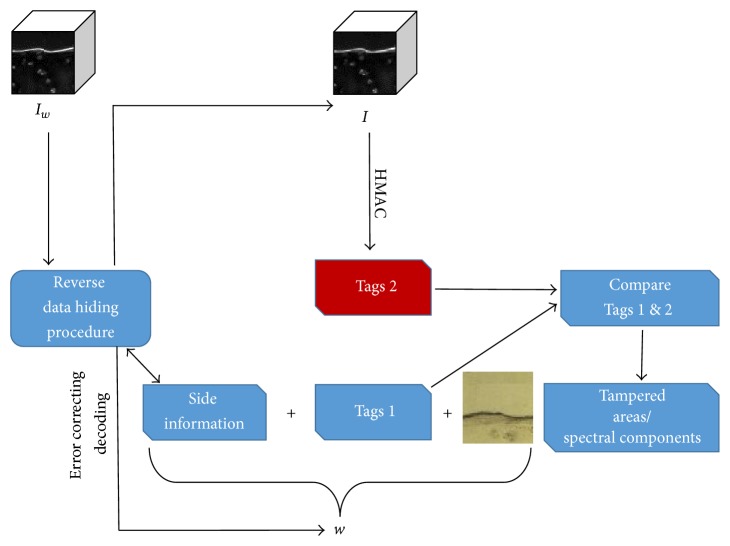
Extracting the payload *w* from the host FTIR image *I*_*w*_.

The payload *w* is extracted and decoded from the FTIR image *I*_*w*_ which is reversed to the initial state *I*. Possible tampering in *I*_*w*_ can be indicated comparing the authentication tags contained in *w* with tags generated from the reversed *I*. The procedure is demonstrated in the block diagram of Figure 3.

Using the double histogram shifting approach with the modification described in Section 3.4 capacity is high and good fidelity between the original FTIR image and the one containing hidden data can be achieved. Moreover and most importantly, the host is reliably reversed to its original state. Furthermore, we demonstrate an alternative use of histogram shifting approaches as we did in [29] where we were using 32-bit MREG based on an approach initially designed for 8-bit static images. In this case, we use 24-bit FTIR images.

For the attack scenario of bit flipping by increasing or decreasing values of one or more spectral components of pixels by one unit, Reed-Solomon codes were selected. For each component of the payload, Reed-Solomon* n* and* k* had different values because of the different characteristics of the data. Furthermore, those were also dynamically selected when the side information was encoded, depending on the available capacity of the current pair of spectral components. Concerning the visible light image, the settings were different for the five most significant bits compared to the remaining three to make sure that the important information remains intact. Having the same settings for all bits would not be possible as the available capacity is not enough. Settings concerning authentication tags were always fixed. The exact *n* and *k* values used in each of the above cases will be stated in the step by step algorithmic description of Section 4.2.

Last, concerning the authentication tags, the HMAC-SHA256 is used to create two separate groups of tags out of the input FTIR image. The first group is created out of sets of spectral components to later determine the wavelength where tampering has taken place. In our implementation, we have used one authentication tag for every set of consecutive five spectral components. The second group of authentication tags is generated out of spatial areas to determine the pixels that have undergone tampering. In our case, we used tags for every 8 × 8 block of pixels. This will allow a double level description of the tampered area including both spatial and spectral information. The tags which were generated from the original FTIR image and now extracted with the rest of the payload, compared to the ones generated from the reversed FTIR image can show areas that failed to get to their original state or had modifications because of attacks. That is done indicating those ranges of five spectral components that contain the error and the 8 × 8 block of pixels where this occurred.

### 4.2. Embedding Procedure

**Figure 4:**
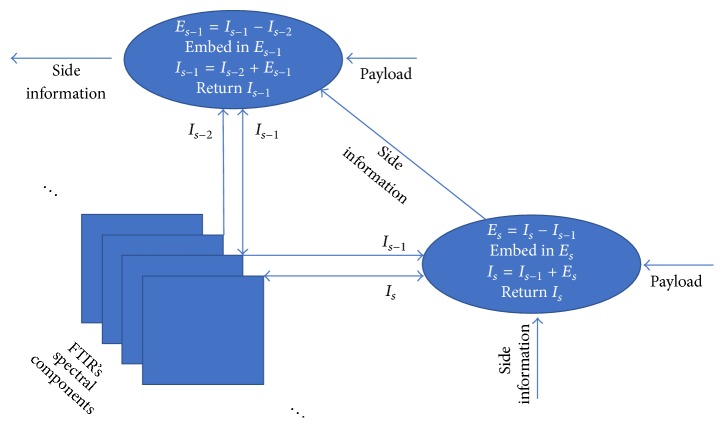
Demonstration of the embedding procedure by accessing spectral components in reverse order.

The embedding procedure embeds data by accessing pairs of consecutive spectral components in reverse order, starting from (1555,1556) and ending in (1,2). This procedure is demonstrated in the diagram of Figure 4. In every iteration, the two peak and zero points of the histograms are converted to binary form and this side information is embedded in the next iteration. When the first spectral component is reached, the peak and zero points for the last pair of spectral components that were accessed along with other possible bits of payload are stored with another reversible method for 2D images, for instance, Tsai et al.'s method [25], or even another method that does not require side information.

Step by step, the embedding procedure works as follows.

*Input Step*

- Read FTIR image *I* of size *M* × *N* and *L* spectral components.
- Read visible light image *V* of size *X* × *Y*.

*Step 1*

- Convert *V* to binary.
- Encode *V* using Reed-Solomon (*n* ← 31, *k* ← 3 for 5 most significant bits, *n* ← 7, *k* ← 3 for 3 least significant bits).
- For every set of 5 spectral components
    generate authentication tags.
- For every 8 × 8 block of pixels
    generate authentication tags.
- Encode all tags using Reed-Solomon (*n* ← 255, *k* ← 32).
- Encode all possible additional payload using Reed-Solomon; *n* and *k* values depend on the format of the input data and the available capacity.
- Combine everything in a single payload *w*.
- * index *←* 1.*

*Step 2*

**Figure 5:**
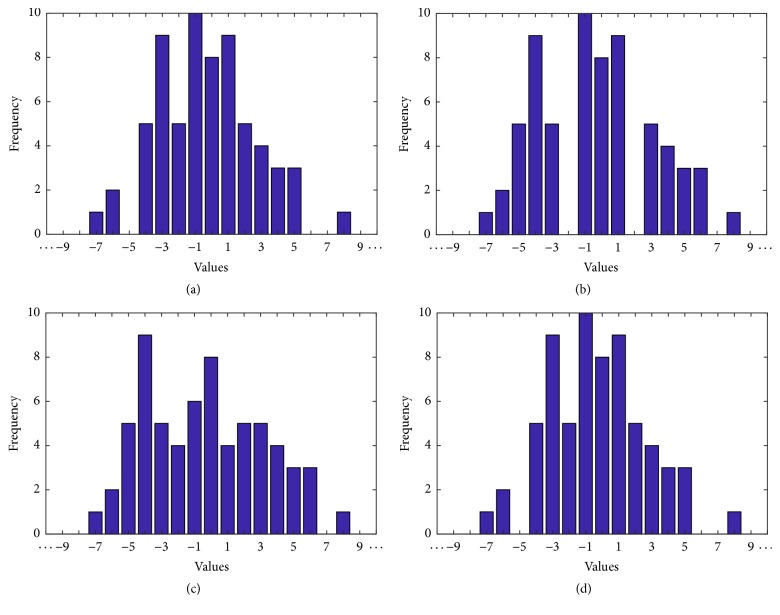
(a) Original histogram. (b) Shifted histogram. (c) Histogram with payload. (d) Reversed histogram.

  Initiate Loop *L* ≥ *s* ≥ 2, *s* ← *s* − 1:
  - Calculate spectral component difference (see (2))
  - *E*_*s*_ ← *I*(1 : *M*, 1 : *N*, *s*) − *I*(1 : *M*, 1 : *N*, *s* − 1).
  - Calculate quantization step *Q* (see (3)).
  - Using step *Q* calculate *E*_*s*_ values' threshold matrix *Qp*, thresholds will show the range of values that correspond to each particular histogram bin. $\underset{}{\max}v$ and $\underset{}{\min}v$ are the maximum and minimum values in *I*;
  - *c* ← 1
  - for $\underset{}{\min}v-\underset{}{\max}v\leq i\leq \underset{}{\max}v-\underset{}{\min}v$, *i* ← *i* + *Q*
  -     *Qp*(*c*)⟵*i*,
  -     *c*⟵*c* + 1.
  - Calculate histogram *hq* based on *Qp* boundaries.
  - Calculate left peak and zero points and left peak and zero indexes *Lmx*, *Lmn*, *Lmxi*, *Lmni*. In histogram (a) of Figure 5 those are 10, 0, −1, and −5, respectively.
  - Calculate right peak and zero points and right peak and zero indexes* Rmx*,* Rmn*,* Rmxi*,* Rmni*. In histogram (a) of Figure 5 those are 9, 0, 1, and 6, respectively.
  - * capacity *←* Rmx* +* Lmx.*
  - From second iteration onwards
  - if *s* < *L*
    1. if* capacity* > 2040
        encode* ws* using Reed-Solomon (*n* ← 255, *k* ← 8)
    2. else if* capacity* > 378
        encode *ws* using Reed-Solomon (*n* ← 63, *k* ← 11)
    3. else
        encode *ws* using Reed-Solomon (*n* ← 31, *k* ← 13).
  - Payload* wcur* to be embedded in this loop is a concatenation of *ws* and *w (index: index* +* capacity* −* size*(*ws*) − 1); *ws* value is from the previous iteration; thus if *s* = *L* then *ws* is empty.
  - * index *←* index* +* capacity.*
  - Convert current [*Lmxi*, *Lmni*, *Rmxi*, *Rmni*] to binary and store in *ws*.

*Step 3*. Do histogram shifting operations accessing all *E*_*s*_ values. Bins following the left zero up before the peak location, that is, *hq*(*x*), for every *x* ∈ [*Lmni*, *Lmxi*) are shifted to the left by one histogram unit creating one empty bin next to the maximum peak location's bin. From the right side, it will be bins following the right peak point until the right zero point, that is, *hq*(*x*), for every *x* ∈ (*Rxi*, *Rmni*] shifted to the right. The process is also illustrated in Figure 5.

     For 1 ≤ *x* ≤ *M*, 1 ≤ *y* ≤ *N*
    (i) if *Qp*(*Lmxi*) > *E*_*s*_(*x*, *y*)
        if *Qp*(*Lmni*) ≤ *E*_*s*_(*x*, *y*)
          *E*_*s*_(*x*, *y*) ← *E*_*s*_(*x*, *y*) − *Q*;
    (ii) if *Qp*(*Rmxi* + 1) ≤ *E*_*s*_(*x*, *y*)
        if *Qp*(*Rmni* + 1) > *E*_*s*_(*x*, *y*)
          *E*_*s*_(*x*, *y*) ← *E*_*s*_(*x*, *y*) + *Q*.

*Step 4*. The algorithm accesses the* Es*'s values that fall in the range, which corresponds to the peak location histogram bins. Value indexes in this range are returned sequentially using function *GetNextIdx*( ). As the binary information to be embedded is accessed sequentially, the voxel in order maintains its value to store a bit of 0 or is increased or decreased depending on whether it is the right or left peak, respectively, to hide a bit of 1. The modified difference matrix is added to the spectral component in index *s* − 1 to create the new spectral component in index *s*, as illustrated in Figure 4. Note that when the algorithm reaches the first spectral component, an alternative method for 2D images is employed. The result of this operation is demonstrated in histogram c of Figure 5.

    (i) For 1 ≤* id* ≤* capacity*
    (1) (*xi*, *yi*, *position*)*← GetNextIdx*(*Lmxi*, *Rmxi*);
    (2) if *wcur*(*id*) = 1
      (a) if* position* =* Left*
          *E*_*s*_(*xi*, *yi*) ← *E*_*s*_(*xi*, *y*) − *Q*
      (b) if* position* =* Right*
          *E*_*s*_(*xi*, *yi*) ← *E*_*s*_(*xi*, *y*) + *Q*.
    (ii) *I*_*w*_(1 : *M*, 1 : *N*, *s*) ← *I*(1 : *M*, 1 : *N*, *s* − 1) + *E*_*s*_ (see (2)).
  Use an alternative method to store the peak and zero points for the two last spectral components hiding it in *I*_*w*_(1 : *M*, 1 : *N*, 1).

*Output Step*

  Return *I*_*w*_.

### 4.3. Extracting/Recovering Procedure

The extracting procedure extracts hidden data and reverses the image by accessing pairs of consecutive spectral components in increasing order, starting from (1,2) and ending in (1555,1556). In every iteration, the peak and zero point for the next pair are obtained from the extracted binary stream. Once again, concerning the first pair, peak and zero points are extracted from the first spectral component using an alternative method. Step by step, the procedure performs as follows.

*Input Step*

  Read FTIR image *I*_*w*_ of size *M* × *N* and *L* spectral components.

*Step 1*

- *w* ← [].
- Extract the initial [*Lmxi*, *Lmni*, *Rmxi*, *Rmni*] from *I*_*w*_(1 : *M*, 1 : *N*, 1) and recover *I*(1 : *M*, 1 : *N*, 1).
- Initiate Loop 1 ≤ *s* ≤ 1555, *s* ← *s* + 1:
-  (1) Calculate spectral component difference
-    *E*_*s*_ ← *I*_*w*_(1 : *M*, 1 : *N*, *s* + 1) − *I*(1 : *M*, 1 : *N*, *s*).
-  (2) Calculate *Q*, *Qp*, *hq* (see, embedding Step 2).
-  (3) Capacity ←*qh*(*Lmaxi*) + *qh*(*Lmaxi* − 1) + *qh*(*Rmaxi*) + *qh*(*Rmaxi* + 1).

*Step 2*. The algorithm accesses the* E*_*s*_'s values which have intensity value within the range which corresponds to the peak location histogram bins, including the ones next to them which resulted from the modified values during embedding. Values in this range are returned sequentially using function* GetNextIdx*(). The ones that retained their values extract 0 bits, while the modified ones extract 1 bit. In the meantime, values are modified back to the peak points.

  (i) *wcur* ← [].
  (ii) For 1 ≤* id *≤* capacity*
    (1) (*xi*, *yi*, *position*) ← *GetNextIdx*(*Lmxi*, *Lmxi* − 1, *Rmxi*, *Rmxi* + 1).
    (2) If* position* =* Left*
      (a) if *Qp*(*Lmxi* + 1) > *E*_*s*_(*xi*, *yi*)
          if *Qp*(*Lmxi*) ≤ *E*_*s*_(*xi*, *yi*)
             *wcur*(*id*) ← 0
      (b) if *Qp*(*Lmxi*) > *E*_*s*_(*xi*, *yi*)
          if *Qp*(*Lmxi* − 1) ≤ *E*_*s*_(*xi*, *yi*)
             (I) *wcur*(*id*) ← 1
             (II) *E*_*s*_(*xi*, *yi*) ← *E*_*s*_(*xi*, *yi*) + *Q*.
    (3) If* position* =* Right*
      (a) if *Qp*(*Rmxi* + 1) > *E*_*s*_(*xi*, *yi*)
          if *Qp*(*Rmxi*) ≤ *E*_*s*_(*xi*, *yi*)
             *wcur*(*id*) ← 0
      (b) if *Qp*(*Rmxi* + 2) > *E*_*s*_(*xi*, *yi*)
          if *Qp*(*Rmxi* + 1) ≤ *E*_*s*_(*xi*, *yi*)
              (I) *wcur*(*id*) ← 1
              (II) *E*_*s*_(*xi*, *yi*) ← *E*_*s*_(*xi*, *yi*) − *Q*.

*Step 3*. Do the reverse histogram shifting operations accessing all *E*_*s*_ values. Bins following the left zero location up before the peak location, that is, *hq*(*x*), for every *x* ∈ [*Lmni*, *Lmxi*) are shifted to the right by one histogram unit. From the right side of the histogram, bins following the right peak point until the right zero point, that is, *hq*(*x*), for every *x* ∈ (*Rxi*, *Rmni*] will shift to the left. The end result is demonstrated in histogram *d* of Figure 5. In order to reverse the spectral component *s* + 1 of the image the components are added with the difference matrix *E*_*s*_.

  (i) For 1 ≤ *x* ≤ *M*, 1 ≤ *y* ≤ *N*
    (1) if *Qp*(*Lmxi*) > *E*_*s*_(*x*, *y*)
      if *Qp*(*Lmni*) ≤ *E*_*s*_(*x*, *y*)
       
*E*_*s*_(*x*, *y*) ← *E*_*s*_(*x*, *y*) + *Q*
    (2) if *Qp*(*Rmxi* + 1) ≤ *E*_*s*_(*x*, *y*)
      if *Qp*(*Rmni* + 1) > *E*_*s*_(*x*, *y*)
       
*E*_*s*_(*x*, *y*) ← *E*_*s*_(*x*, *y*) − *Q*.
  (ii) Get the image payload data and add it in *w* in front of the data that it already contains. Side information is decoded with the Reed-Salomon Codes. *n* and *k* will be the same as in embedding. Decoded data is converted to decimal and stored in [*Lmxi*, *Lmni*, *Rmxi*, *Rmni*].
  (iii) *I*(1 : *M*, 1 : *N*, *s* + 1) ← *I*(1 : *M*, 1 : *N*, *s*) + *E*_*s*_ (see (2)).

*Step 4*

- In the reverse way to embedding Step 1 all the information is decoded from *w*, and thus the image *V* is acquired, as well as all authentication tags.
- For every set of 5 spectral components
    generate authentication tag from *I* and compare with extracted tag and indicate possible tampered data if they are not equal.
- For every 8 × 8 block of pixels
    generate authentication tag from *I* and compare with extracted tag and indicate possible tampered data if they are not equal.

*Output Step*

  Return *I*, *V*.

### 4.4. Removal and Cropping Attack Scenario

Additionally, to Reed-Solomon codes, we studied and verified the effectiveness of deletion channel correction codes for robustness against removal and cropping attacks; the method remains the same but every application of Reed-Solomon codes is now replaced by deletion channel correction codes. Those are applied as described in Section 3.3. Unlike Reed-Solomon codes which had varying properties, in this case, the properties of this deletion channel correction codes were identical for all the different components of the payload. Specifically, those were seven redundancy bits per byte.

Note that in this case there is a generated key for each single use of the deletion channel error correcting codes. Those keys are stored in a file and used as input for decoding during the extracting/reversing procedure. Thus, unlike the default version where the only side information required is for the generation of the authentication tags, in here, side information is required for error corrections.

## 5. Results and Discussion

For the experimental purposes, FTIR image and visible light image samples have been collected from our university hospital following research procedures with informed consent. All the FTIR image samples were of size 64 × 64, including 1,556 spectral components and a bit depth of 24 bits. The embedded visible light images were 100 × 100 RGB image blocks with a bit depth of 8 bits. Seven different attack test scenarios with different characteristics were studied. Every test was repeated five times using different data each time, that is, different pairs of FTIR images and visible light images. Firstly, the functionality of the method was tested by running it without applying any attacks, to verify reversibility. Secondly, in order to test robustness and tamper detection, there were three different bit flipping attacks and four cropping/removal attacks. More details will follow in Section 5.4.

### 5.1. Reversibility

Prior to attacks, on the host FTIR image, we confirmed reversibility by extracting/reversing an intact FTIR image that contained hidden data. In this test, the payload image was extracted intact, but most importantly, the authentication tags did not indicate any possibly tampered areas or tampered spectral components, proving that the method extracted the payload successfully but also reversed to its exact initial state. That was also confirmed by manually operating a simple subtraction between the original FTIR image and the reversed one.

### 5.2. Capacity and Fidelity

Capacity depends on the type of error correcting codes that were utilized for the different attack scenarios. This is because the side information size is different, depending on whether Reed-Solomon or deletion channel correction encoding is applied. The following capacity results include the mean capacity and the standard deviation from five repetitions with different data.

Beginning with the use of Reed-Solomon codes by running five different sample images, full capacity is 5,842,880 ± 34,320 bits, while capacity with the side information subtracted is 2,758,483 ± 32,613 bits. The encoded payload visible light image's size is 1,760,000 bits and the authentication tags' size is 765,000 bits.

In the second case where deletion channel correction codes are employed for robustness against cropping and removal attacks, full capacity remained the same but the real capacity after subtracting the side information is 5,051,443 ± 34,160 bits. This time, the size of the encoded visible light image is 1,920,000 bits, while the size of the authentication tags is 768,000 bits.

**Figure 6:**
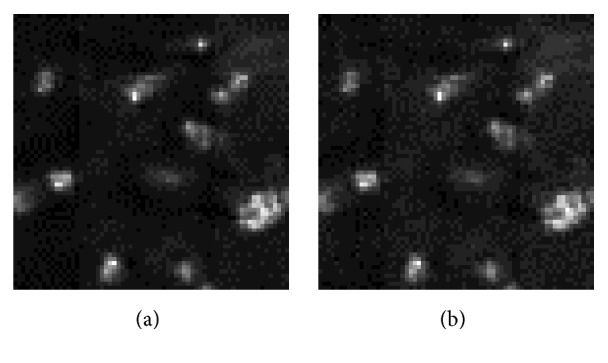
(a) Original FTIR image's spectral component (2900 cm^−1^). (b) The same spectral component (2900 cm^−1^) after data has been hidden.

**Figure 7:**
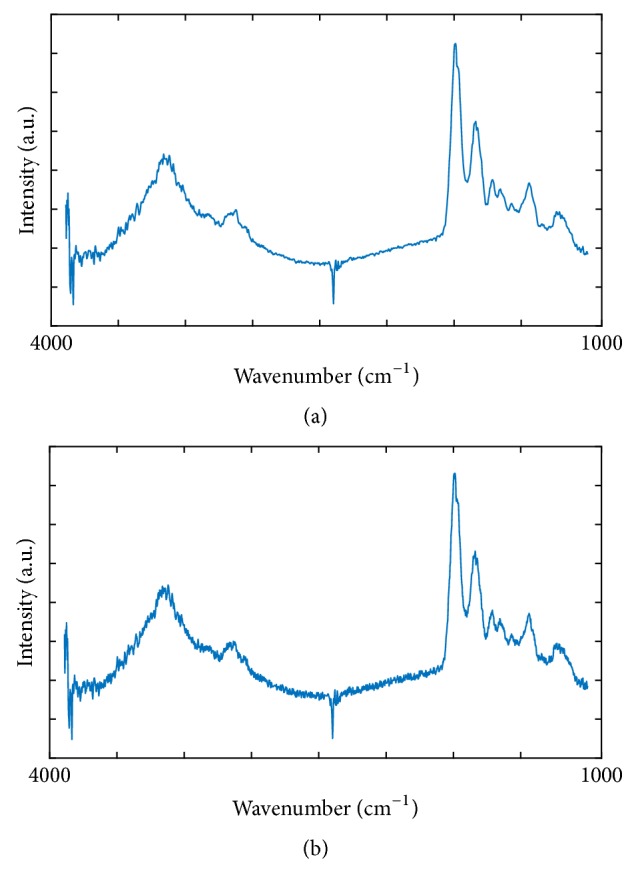
(a) Original FTIR spectrum from a single pixel. (b) Spectrum from the same pixel after the data has been hidden.

Fidelity was good as the FTIR image containing hidden data was very close to the original one. Specifically, Peak Signal to Noise Ratio (PSNR) values using five sample images were 34.4 ± 1.5 dB when Reed-Solomon codes were employed. As a demonstration, a single spectral component before and after data hiding is shown in Figure 6.  Figure 7 similarly compares spectra out of a single pixel acquired from the same image. Then, PSNR was 34.8 ± 2.2 dB when the deletion channel error correction coding was employed for the same five data samples.

### 5.3. Tamper Detection

The data hiding method offers tamper detection through the comparison of authentication tags generated from the original FTIR image using HMAC-SHA256 and embedded in the FTIR image with tags generated from the reversed FTIR image after the payload has been extracted.

Specifically, tamper detection is realized by comparing the authentication tag produced of each set of five consecutive spectral components but also for each set of 8 × 8 blocks of image pixels. For instance, the tamper detection function could output that there is a possibility of tampering in spectral components 350–354 and the block of pixels with coordinates [9-16]×[1-8] and thus that part is not safe to use in a diagnosis. Note that, in every experiment performed to check robustness, the extracted authentication tags indicated successfully tampered FTIR image locations. Since HMAC-SHA256 was used, even a single error in a spectral component of one pixel is able to trigger positive indication for a tampered 8 × 8 block and a group of 5 spectral components that contains it. However, even a single error in the extracted and decoded stream that represents the authentication tags can result in false positive alerts about tampering. Of course, false positives are of secondary priority and what was significant here is that there were never cases of false negative alerts about tampering. That ensured that in every case that the FTIR image contains errors it is always indicated successfully and it is not used in any vital analyses. More specific details about those errors follow in the next subsection.

### 5.4. Robustness

The following experiments were performed to test all possible attacks described in Table 1. They were run with the FTIR image after the embedding procedure and before running the extracting and recovery procedures. Those experiments tested in which level the error correcting codes succeed in recovering the payload, how accurately the FTIR image is reversed, and how accurately the authentication tags show tampered areas. There were again five repetitions for each one of the seven experiments using different data each time. Error probability refers to the probability of introducing error to each individual spectral component of each pixel in the image, that is, each individual cell in the 3D data structure. The modified values refer to the number of actual values that were modified by the attack. Last, error correction refers to the error correcting code that was used in each case.

Experiments 1 to 3 had bit flipping in the LSBs of the FTIR image with 0.01% and 1% probabilities. That was done by increasing or decreasing intensity values by one unit each time with the corresponding probability. As for experiments 4 to 7, removal attacks were applied. More specifically, experiments 4 and 5 had random value removal with 0.001% and 0.01% probabilities, while experiment 6 had all spectral components removed for one specific FTIR image point pixel and last experiment 7 had 3 × 3 rectangles of pixels cropped from 4 separate spectral components. Experiments 1 to 3 used Reed-Solomon codes, while experiments 4–7 used deletion channel correction encoding because cropping attacks are more likely to cause missing bits other than flipped bits.

Beginning with the first component of the payload, that is, the authentication tags, certain cases included errors resulting in false positives, as already described in Section 5.3. Specifically, experiments 2–7 did contain false positives as alerts were shown for other areas except the ones that were indeed attacks. Experiment 1 indicated successfully only the attacked areas, and of course, in the scenario when no attacks were applied at all, authentication tags did not show any alerts.

Another component inside the payload was the side information which enabled the selected histogram shifting data hiding method to run successfully. In this case, there was zero error tolerance and that is the reason why the solution presented in Section 3.3 was integrated. The success ratio here was significantly high. Specifically, thanks to the error correcting solutions, out of the 35 test runs that included attacks, 34 managed to retrieve and recover the side information making the extracting procedure run successfully. There was only one failure in experiment number 5 after an error occurred in the side information of one iteration and the operation halted.

**Figure 8:**
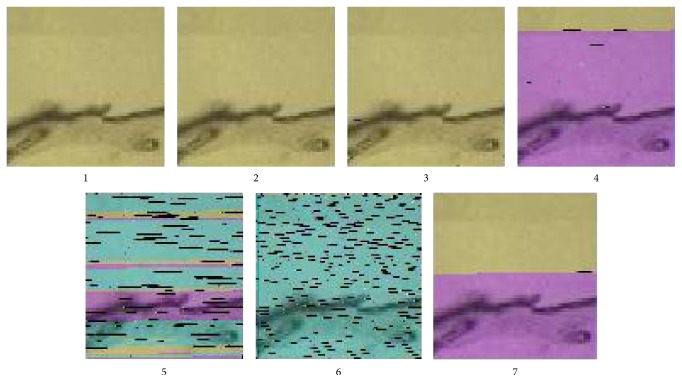
The extracted visible light image from 7 experiments.

Table 2 includes further information on the result of every experiment. The “payload image” column describes the state of the extracted visible light image payload reporting the PSNR values comparing them with the original images. In experiments 1 to 3, there were hardly any noticeable artifacts, while in experiments 4 to 7, there were color distortions due to bit shifts caused by missing bits. Still those errors can be tolerated as the visible light images are not used in the analyses but they merely assist by indicating the location of the tissue from which the FTIR image originates. The image texture is enough to indicate location. The second column shows the amount of FTIR image values that had errors. In every case, modifications caused by the hidden data were removed and that is why the errors were highly correlated to the number of errors inserted through attacks. An exception usually appears in neighboring to removed values and that is why intensity errors tend to increase when deletion error probability reaches 0.01% in experiment 5. It should be further noticed that 2 and 3 intact images were extracted from experiment number 2 and number 3, respectively, and there was a single failure in experiment number 5.  Figure 8 demonstrates the state of the extracted visible light image acquired during the first experiment run.

### 5.5. Discussion

As it has been demonstrated by the experimental results, the presented method is able to hide data with high capacity and good fidelity and reliably extract it and reverse the FTIR image in its initial state to make it safe for analyses. In case of attacks or modifications in FTIR images, the authentication tags will indicate possibly tampered areas and thus analyses can then be avoided from those locations.

High capacity was necessary for hiding visible light image data. Furthermore, error correcting codes multiply the needs for capacity. Thus, this was the reason that a double histogram shifting, that is, using two histogram point pairs, on difference matrices was proposed as the data hiding scheme. The choice of two histogram pairs combined with its application on the difference matrix and the use of quantization comes with a cost to fidelity in favor of capacity. However, it is demonstrated in the experimental results that fidelity was enough to make the FTIR images perfectly acceptable for viewing. For the analyses' purposes, the images are reversed to their initial state and thus the level of modifications in the images that contain hidden data does not have any effect on the diagnoses. For reference, experimental results showed that, using the above scheme, the current approach offered an average pure capacity of 0.92 bits/value, where value stands for the pixel of spectral component unit. After subtraction of side information, the capacity was 0.43 bits/value and 0.79 bits/value with the use of Reed-Solomon and deletion channel correction codes, respectively. As for the fidelity between the original FTIR image and the one containing hidden data, maintaining the above capacity, the PSNR was over 34 dB.

Since the method was based on difference matrices of spectral components and FTIR images have higher bit depth and are overall different, there can be no direct comparison with the other histogram shifting schemes. However, some information can be acquired by comparing the capacity per unit and PSNR. Ni et al. [24] reported a capacity of about 5–80 kb tested on various 512 × 512 images, which is 0.02–0.31 bits/pixel. The PSNR was over 48 dB. An improvement followed by Tsai et al. [25]. In their proposal, the capacity varies depending on the set of parameters. To give some examples, using 3 × 3 prediction blocks and two histogram nonoverlapping pairs similar to the proposed technique there was an average capacity of 0.25 bits/pixel and a PSNR of 49.85 dB. Even higher capacity was obtained with three overlapping pairs, reaching an average of 0.47 bits/pixel and a PSNR of 43.61 dB. Another improved method based on histogram shifting was proposed by Fallahpour et al. [26]. The method improved capacity by 30% to 200% and maintained good fidelity by employing image tiling in blocks before histogram shifting operations. Results from an example showed that capacity is 0.04–0.14 bits/pixel when PSNR is ranging between 40 dB and 52 dB. Last, to compare with a different approach, Tian [30] proposed a reversible data hiding paper based on difference expansion exploring the redundancy of pixels. Using different settings the capacity starts from 0.15 bits/pixel and a PSNR of 44.30 dB up to 1.97 bits/pixel and a PSNR of 16.47 dB. It should be also noted that the capacity of 0.67 bits/pixel is achieved with a PSNR of 34.80 dB.

In the first attack scenario, Reed-Solomon codes were able to extract the payload with high precision and the false positives from the extracted authentication tags were limited. Errors in the extracted visible light image were more prevalent in cropping/removal attacks and the employment of deletion channel correcting codes, although most of those errors were shifts due to missing bits; thus the content of the image was visible but the images colors distorted. The point where this problem was more significant was at the extraction of the authentication tags. Tags were able to detect that there were errors in the FTIR image but they could not indicate only the tampered areas as they also revealed that the whole or most of the FTIR image structure was tampered. This is an issue that can be fixed with a solution similar, for instance, to the one applied for the side information, as described in Section 3.3. What made it practically infeasible to apply for the visible light image but also for the authentication tags was the fact that it was a considerably time-consuming process since it was based on a trial and error approach. The issue can be approached with two options: the application of the same solution proposed for side information in cases with no time limitations and the development of a highly optimized version of the current method.

It was shown that by comparing authentication tags the FTIR image can be reversed successfully and that there are no error artifacts in the spectral or spatial domain. It should be always taken into account, however, that there might be some false negatives since errors might be present in the extracted authentication tags, which will indicate the unlikely event of failed reversion in a specific area with that area having been completely recovered.

## 6. Conclusions

This paper presented a method for hiding visible light images or other information such as EPR in FTIR images with a main purpose of providing efficiency in data management and storage. Furthermore, the paper addressed security issues, that is, confidentiality, availability, and reliability of content. Tamper proofing and recoverability capabilities are additionally provided and small alterations on the host can be detected. The data hiding method can guarantee with the proper quantization settings high capacity and good fidelity between the original FTIR structure and its version that carries hidden data. Moreover, reversibility is available. Along extraction of data, the method reverses the FTIR image that carries hidden data to its original state. Last, two different approaches for error correction were suggested to enable corrections of the payload data after extraction.

What shall be researched in future work is a more optimized method for error correction. It was noted that bit errors cause problems in deletion channel correction encoding. The solution presented here is time-consuming and thus it was used only for the part of the payload created out of the side information which was of the highest priority. Specifically, with the current setup this solution added ~40 sec delay in the iterations where bit errors were detected. Improvements in here would allow great quality increase in the extracted visible light images, as well as the precision of tamper detection.

## Figures and Tables

**Table 1:** FTIR image attacks.

#	Error probability	Modified values	Error correction
1	0.01%	626 ± 32	Reed-Solomon
2	0.1%	6381 ± 91	Reed-Solomon
3	1%	63863 ± 22	Reed-Solomon
4	0.001%	60 ± 4	Deletion channel
5	0.01%	658 ± 27	Deletion channel
6	0.025%	1556	Deletion channel
7	0.0004%	27	Deletion channel

**Table 2:** Payload and FTIR images' recoverability after attacks.

#	Payload image (dB)	Intensity value errors (spectral component pixels)
1	Intact	626 ± 32
2	42 ± 0.0	6355 ± 62
3	37.5 ± 0.7	63865 ± 216
4	21.4 ± 8.3	167 ± 23
5	11.8 ± 0.5	2166 ± 824
6	11 ± 1.7	1553 ± 7
7	18.6 ± 5.8	165 ± 13
